# Assessment of serum sialic acid correlated with C3 in children with *Mycoplasma pneumoniae* pneumonia

**DOI:** 10.1002/jcla.23078

**Published:** 2020-01-06

**Authors:** Yu‐Mei Mi, Qi Qi, Li Zhang, Xiao‐Fang Wang, Zhi‐Min Chen, Chun‐Zhen Hua

**Affiliations:** ^1^ Division of Infectious Disease Children's Hospital Zhejiang University School of Medicine National Clinical Research Center for Child Health Hangzhou Zhejiang China; ^2^ Department of Infectious Disease Hangzhou Red Cross Hospital Hangzhou Zhejiang China; ^3^ Department of Pediatrics The Affiliated Hospital of Hangzhou Normal University Hangzhou China; ^4^ Division of Respiration Children's Hospital Zhejiang University School of Medicine Hangzhou Zhejiang China

**Keywords:** C3, children, *Mycoplasma pneumoniae*, pneumonia, sialic acid

## Abstract

**Background:**

Different from the diagnosis of bacterial infections, *Mycoplasma pneumoniae* pneumonia (MPP) is still lacking of convenient non‐specific laboratory parameters.

**Method:**

A total of 125 children with MPP were included in the MPP group and 89 children with *Mycoplasma‐*negative pneumonia were included in the control group, and the sera were collected from the children at both the acute and recovery stages in the two groups.

**Results:**

The sialic acid and C3 in the MPP group were significantly higher than those in the control group both at the acute and at the recovery stage. On the other hand, the sialic acid and C3 at the acute stage were significantly higher than those at the recovery stage in the MPP group. However, in the control group, the sialic acid and C3 demonstrated IgG exhibited no significant change between the acute stage and the recovery stage. Lastly, positive correlations between sialic acid level and C3 level were identified in the MPP group at both acute and recovery stages.

**Conclusion:**

Our study demonstrated that the serum sialic acid correlated with C3 specifically increased in children with MPP, indicating that it might be the important non‐specific parameters in the diagnosis of MPP.

## 1 INTRODUCTION

*Mycoplasma pneumoniae* pneumonia (MPP) is a common kind of community‐acquired pneumonia, which can lead to long‐duration, prolonged fever, repeating cough, anhelation, and even extrapulmonary organ damage, and other symptoms. The pathophysiologic mechanisms of MPP include direct damage caused by pathogens and indirect damage caused by immune response.^1^, ^2^, ^3^ Current guidelines recommend PCR and single‐sample serological tests to diagnose Mycoplasma pneumoniae infections. The “gold standard” for diagnosis of MPP is still considered to be a fourfold increase in antibody titer as measured in paired sera.^4^ Additionally, other methods provide important support for the diagnosis of mycoplasma pneumoniae pneumonia, such as using PCR to detect MP‐DNA or RNA.^5^, ^6^

Sialic acid is a derivative of neuraminic acid, a 9‐carbon keto sugar, that occupies the interface in some cells between the host and commensal/pathogenic microorganisms.^7^, ^8^ It was reported that sialic acid is usually located in the glycoproteins and glycolipids, and exhibits anti‐inflammatory effects.^9^ Currently, the clinical research of serum sialic acid is focused on the field of cancer. Sialic acid has been identified as a tumor‐associated antigen, which is overexpressed on cell surface and reveals the malignant and metastatic phenotypes for various types of cancers.^10^, ^11^ Moreover, the tumor cells avoid the host immune response by the surface antigen by sialylation.^10^ Therefore, increased levels of serum sialic acid could be an important marker in diagnosis of malignancy tumors.

Different from the diagnosis of bacterial infections, which can be diagnosed by experimental assays, such as routine blood test, CRP, and procalcitonin, MPP is still lacking of convenient non‐specific laboratory parameters. Hence, in this study, we explored the possibilities of sialic acid and complement 3 (C3) as the important non‐specific parameter in the diagnosis of MPP. As we all know, there is rich amount of sialic acid on some serum protein's surface, such as complement components and binding globulin. *Mycoplasma pneumoniae* infection could lead to the body's immune function change.^1^, ^2^, ^3^ Thus, we speculated that there may be corresponding changes between serum sialic acid level and complements in MPP children. This study is intended to discuss the diagnostic importance of some non‐specific parameters in MPP by analyzing the levels of serum sialic acid, immunoglobulin G (IgG), C3, or C4, and their correlations.

## 2 MATERIALS AND METHODS

### 2.1 Subjects

The children diagnosed with pneumonia and admitted in Hangzhou Red Cross Hospital from July 2011 to June 2013 have been enrolled in this study. The MPP group included cases that are consistent with the following two conditions: (a) Meet the pneumonia's diagnostic criteria and (b) the serum MP‐IgG in recovery time increased more than four times in the acute phase, or accompanied by MP‐DNA positive in sputum or throat swab. The control group included cases that were consistent with pneumonia's diagnostic criteria, but without increasing of MPP‐IgG or MP‐DNA positive in sputum or throat swab. This study was approved by the ethics committee of Hangzhou Red Cross Hospital, and the study protocol conforms to the ethical guidelines of the 1975 Declaration of Helsinki. All patients signed written informed consent.

### 2.2 Reagents and instruments

Sialic acid assay kits were bought from the Beijing Jiu Qiang Biotechnology Co. Ltd (production license number is 20020023). The kits for C3, C4, IgG (production batch: 67839, 67871, 67731) were bought from Finland Orion Diagnostica Company. The other equipments in this study include automatic biochemical analyzer type AU 5400 from Japan's Olympus, type MDF‐382E of ultra‐low temperature refrigerator from Dirui CS‐400B automatic chemical analyzer for detecting Ig, C3, and C4.

### 2.3 Methods

The samples about 300 μL of separated plasma were taken on their admission day and their recovery period, respectively. Then, the samples were stored in the refrigerator at −70℃ for later use. Sialic acid was detected by assay of neuraminidase enzyme.^10^ C3 and C4 were detected by using turbidimetric immunoassay. IgG was detected by using passive agglutination assay.

### 2.4 Statistical analysis

The analysis of data was carried out using SPSS version 17.0, the difference between groups was analyzed using Student's *t* test for all statistical analysis, and values were expressed as mean ± SD. All data types were normal distribution. The significance level was set at *P < *.05. The Pearson correlation analysis was used for correlation.

## 3 RESULTS

### 3.1 General data

A total of 125 cases with MPP were included in the MPP groups, having 73 male cases and 52 female cases, with male and female ratio of 1.4:1 (Table 1). The age range was about 11 months to 14 years, with average age of 6.2 ± 3.1 years (Table 1). A total of 89 cases with *Mycoplasma‐*negative pneumonia were included in the control group, with 47 male cases, 42 female cases, and male and female ratio of 1.12:1 (Table 1). The age range of the control group was about 11 months‐14 years, with average age of 5.6 ± 3.1 years (Table 1). The mean days in hospital of MPP group and control group patients were 9.5 ± 2.1 days and 7.5 ± 2.1 days, respectively (Table 1). The mean fever days of MPP group and control group patients were 5.8 ± 3.1 days and 4.9 ± 2.9 days, respectively (Table 1). Lastly, the mean interval days between the detection in the acute stage (A.S) and the recovery stage (R.S) of MPP group and control group were 5.5 ± 1.7 days and 5.0 ± 1.7 days, respectively.

**Table 1:** General information of the control group (89 cases) and MPP group (125 cases)

	Gender			Age (y)	Days in Hospital	Fever days	Days between two detections
	Male	Female	M/F ratio				
Control	47	42	1.12:1	5.6 ± 3.1	7.5 ± 2.1	4.9 ± 2.9	5.0 ± 1.7
MPP	73	52	1.4:1	6.2 ± 3.1	9.5 ± 2.1	5.8 ± 3.1	5.5 ± 1.7

### 3.2 The routine blood test and sCRP

In the control group, the white blood cell, the neutrophilic granulocyte, and sCRP were significantly higher at the acute stage than at the recovery period (*P* < .001) (Table 2). In the MPP group, no differences were found in the white blood cell and the neutrophilic granulocyte between the acute stage and the recovery period, but sCRP level was higher at the acute stage than that at the recovery period (*P* < .001) (Table 2). Compared with the control group, the white blood cell and the neutrophilic granulocyte were lower (*P* < .05) in the MPP group at acute stage (Table 2). However, there were no differences in the white blood cell, the neutrophilic granulocyte, and sCRP between the two groups at recovery phase (*P* > .05) (Table 2).

**Table 2:** Comparisons between the control group (89 cases) and MPP group (125 cases) white blood cell count, neutrophilic granulocyte (NEUT) count, and sCRP in acute phase (A.S) and recovery phase (R.S)

	WBC (×10^9^)				NEUT (×10^9^)				sCRP (mg/L)			
	A.S	R.S	*T*	*P*	A.S	R.S	*T*	*P*	A.S	R.S	*T*	*P*
Control	9.07 ± 4.17	7.25 ± 1.60	3.82	<.001	5.48 ± 3.66	3.43 ± 1.27	4.94	<.001	19.16 ± 3.83	4.54 ± 0.97	3.46	<.001
MPP	7.16 ± 2.71	7.44 ± 2.44	0.63	.53	4.28 ± 2.00	3.86 ± 1.95	1.95	.07	22.02 ± 2.38	5.43 ± 0.92	6.19	<.001
*T*	3.80	0.821	–	–	2.80	1.96	–	–	0.67	0.61	–	–
*P*	0.001	0.413	–	–	0.006	0.052	–	–	0.51	0.55	–	—

### 3.3 The levels of sialic acid, C4, C3, and IgG

In the control group, there was no difference in the level of serum sialic acid between the acute stage and recovery period (*P* > .05), but the level of serum sialic acid was significantly higher in acute phase than that in recovery stage in the MPP group (*P* = .005). Moreover, the levels of serum sialic acid in the MPP group were significantly higher than those in the control group at both of acute and recovery stages (*P* < .05).

In the MPP group, the serum level of IgG at acute stage was lower than that in recovery period (*t* = 2.07, *P* = .04), and the levels of plasma C3 and C4 in acute stage were significantly higher than those in the recovery period (*P* < .05). There was no significant difference in serum IgG and C3 levels between the acute phase and the recovery phase (*P* > .05), but the level of serum C4 in the acute period was significantly higher than that in the recovery period in the control group (*P* = .001). Compared with the control group, the levels of plasma C3 in the MPP group during both at acute and recovery stages were significantly higher (*P* < .05). In recovery stage, the level of C4 in MPP group was lower than that in the control group (*P* = .005), but there was no significant difference in C4 between MPP group and control group (*P* > .05). Lastly, there were no significant differences in IgG between MPP group and control group at both the acute stage and the recover stage. Table 3 shows the comparison of the levels of sialic acid, C3, C4, and IgG in the patients with MPP and the control group.

**Table 3:** Comparisons between the control group (89 cases) and MPP group (125 cases) levels of sialic acid, IgG, C3, and C4 in acute phase (A.S) and recovery phase (R.S)

	Sialic acid (mg/L)				IgG (g/L)				C3 (g/L)				C4 (g/L)			
	A.S	R.S	*T*	*P*	A.S	R.S	*T*	*P*	A.S	R.S	*T*	*P*	A.S	R.S	*T*	*P*
Control	79.2 ± 17.2	77.9 ± 15.3	0.40	.693	8.76 ± 2.92	9.04 ± 2.87	0.65	.52	1.20 ± 0.29	1.17 ± 0.27	0.86	.39	0.31 ± 0.10	0.27 ± 0.09	2.98	.003
MPP	88.0 ± 14.5	83.7 ± 13.9	2.81	.005	9.00 ± 2.68	9.71 ± 2.69	2.07	.04	1.37 ± 0.30	1.26 ± 0.22	3.23	.001	0.34 ± 0.12	0.23 ± 0.10	7.86	<.001
*T*	4.17	2.79	–	–	0.60	2.61	–	–	4.06	2.78	–	–	1.91	2.87	–	–
*P*	<0.001	0.006	–	–	0.548	0.108	–	–	<0.001	0.006	–	–	0.057	0.005	–	–

### 3.4 Correlation analysis

**Figure 1:**
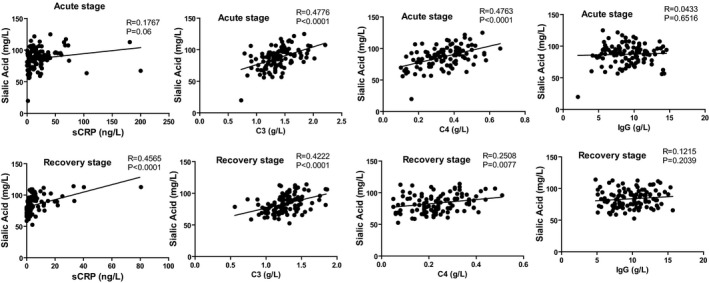
The analysis of the correlations between sialic acid and sCRP, C3, C4, or IgG at the acute and recovery stage in MPP

Analyses of the relationship between the levels of sialic acid and sCRP, IgG, C3, or C4 were performed by the Pearson correlation analysis in MPP group at both acute and recovery stages. The results showed that serum levels of sialic acid and C3 or C4 were significantly positively correlated at both the acute stage and recovery stage (Figure 1). Meanwhile, the sialic acid was significantly positively correlated with sCRP only at the recovery stage (Figure 1).

## 4 DISCUSSION

This study showed that the white blood cell count, neutrophil count, and sCRP in the control group were significantly higher in acute phase compared with the recovery phase in the control group. This could be the effect of antimicrobial agents on bacteria, which implicate that they were mainly suffering from bacterial infection. However, the MPP group had no significant changes in white blood cell count and the neutrophilic granulocyte count, which were significantly lower than those in the control group, while the sCRP at the acute stage was significantly higher than that in the recovery stage. The reasons for the increase in sCRP may be related to the strong immune response toward inflammation of the body in patients with MPP.

The pathogenesis of MPP is very complex. The following mechanisms were currently being discussed, including immunological mechanism of MP infection, the adsorption of airway epithelial cells with hydrogen peroxide damage, and MP direct invasion. At present, the main tendency of immune disorders and autoimmune theories to explain them has attracted much attention of scholars. Cell‐mediated immunity level or predominant response is potentially correlated with the variable pulmonary patterns seen in chest images.^12^, ^13^, ^14^ Multiple studies have acknowledged the importance of IL‐12, interferon‐γ, and Th1‐type T‐cell responses during the course of *M pneumoniae* infections.^15^, ^16^, ^17^, ^18^ In the disease progression, both MP‐specific and non‐specific antibodies are gradually produced, maintaining a high level of IgG until the recovery phase, resulting in increase of total serum IgG level.^19^ In our study, we found that IgG level in recovery phase was significantly higher than that in acute stage in the MPP group, indicating that it might be value for MPP diagnosis.

In other studies, complement system was also indicated to be involved in lung injury. Chest trauma led to elevated expression levels of inflammatory and coagulatory proteins, such as C3.^20^ HIV activates the complement system, predominantly via the classical pathway, and causes increased C4 activation and consumption during sepsis.^21^ Our study showed that the levels of serum C4 and C3 in MPP patients in acute phase were significantly higher than those in the recovery period. Moreover, C3 in the MPP group was higher than that of the control group in acute phase and recovery phase. It is suggested that C3 might be an important non‐specific parameter for MPP diagnosis.

Sialic acid is an important component of the glycoprotein, and it is also closely related to human health and disease. Human blood circulation contains a large number of multifunctional glycoprotein, which are synthesized by liver and then secreted into blood circulation. The sialic acid at the end of the sugar chain can avoid the rapid removal of the glycoprotein from the liver, thereby ensuring the presence of these serum proteins. Till now, few researches on serum sialic acid in infectious diseases could be found. However, the combined detection of sialic acid and sCRP was considered as an important marker for the acute inflammatory reaction.^19^ There are reports showing that the levels of serum sialic acid increased in patients with inflammation, even without any significant difference in patients with tumor.^22^ This study showed that the plasma sialic acid level in acute phase was significantly higher than that in recovery period in the MPP group; however, there was no significant difference in the levels of serum sialic acid in acute phase and recovery phase in patients with *Mycoplasma‐*negative pneumonia. The levels of plasma sialic acid in MPP patients were significantly higher than those in the control group both in acute phase and in recovery phase, which indicated that the sialic acid might be an important non‐specific parameter for MPP diagnosis.

During the process of MPP, the body's synthesis of the acute‐phase reactants, complement protein, and other inflammatory‐associated glycoproteins significantly increased, resulting in a corresponding increase in serum sialic acid levels in children. In our study, the levels of serum sialic acid were positively correlated with the concentrations of C3 or C4, which was consistent with the result that the levels of sialic acid and C3 at the acute stage in the patients with MPP were higher than those in the recovery phase, and also sialic acid and C3 in MPP were significantly higher than those in the control group.

In summary, our study suggested that the routine laboratory blood tests for white blood cells and neutrophil counts, and sCRP, serum sialic acid, and complement component (C3 and C4) levels have certain reference value in diagnosis of MPP. Moreover, our study exhibited that serum sialic acid correlated with C3 at the acute stage was significantly higher than that at the recovery stage in MPP, and serum sialic acid correlated with C3 in MPP was significantly higher than that in *Mycoplasma‐*negative pneumonia at both acute stage and recovery stage, which indicated that serum sialic acid correlated with C3 might be the important non‐specific parameters in the diagnosis of MPP.
